# Increasing the Yield and Cryosurvival of Spermatozoa from Rhinoceros Ejaculates Using the Enzyme Papain

**DOI:** 10.3390/biology11020154

**Published:** 2022-01-18

**Authors:** Jessica P. Rickard, Kelsey Pool, Simon P. de Graaf, Timothy Portas, Natalie Rourke, Miriam Wiesner, Thomas B. Hildebrandt, Frank Göritz, Robert Hermes

**Affiliations:** 1 School of Life and Environmental Sciences, Faculty of Science, The University of Sydney, Sydney, NSW 2006, Australia; kelsey.pool@uwa.edu.au (K.P.); simon.degraaf@sydney.edu.au (S.P.d.G.); 2 Zoo and Wildlife Veterinary Consultancy, Maleny, QLD 4552, Australia; timp@zoovet.com.au; 3 Werribee Open Range Zoo, Werribee, VIC 3030, Australia; nrourke@zoo.org.au; 4 Salzburg Zoo Hellbrunn, 5081 Anif, Austria; mwiesner@salzburg-zoo.at; 5 Department of Reproductive Management, Leibniz Institute for Zoo and Wildlife Research, 10315 Berlin, Germany; hildebrandt@izw-berlin.de (T.B.H.); goeritz@izw-berlin.de (F.G.); hermes@izw-berlin.de (R.H.)

**Keywords:** rhino, viscous, freezing, conservation, flow cytometry, CASA

## Abstract

**Simple Summary:**

Efficient collection and cryosurvival of semen from threatened wildlife species is key for the success of artificial reproductive technologies (ARTs). The viscous nature of ejaculates often collected from species such as rhinoceros, elephant, hippopotamus and primate, render the majority of spermatozoa collected useless and is therefore wasted. The enzyme papain has been used to reduce the viscosity of camelid semen but has yet to be tested in wildlife species. Therefore, the current study investigated the ability of papain to improve the yield and quality of spermatozoa collected from viscous fractions of the rhinoceros ejaculate during cryopreservation. Papain increased the quantity of useable spermatozoa collected from ejaculates, as well as the motility prior to freezing. It also improved the post-thaw motility, velocity, linearity and straightness of samples compared to spermatozoa frozen from the sperm-rich fraction of the ejaculate. There was no detrimental effect on membrane characteristics or DNA integrity. These results show that treating rhinoceros ejaculates with papain is able to salvage valuable spermatozoa and improve survival post-thaw, ultimately increasing the success of ARTs and creation of biobanks for the maintenance and survival of threatened species.

**Abstract:**

The preservation of rhinoceros semen is vital for captive breeding programs. While successful collection and cryopreservation of rhinoceros semen has been reported, the volume and quality of semen produced is often low due to the high viscosity associated with ejaculates collected via electroejaculation. Reducing semen viscosity would enable access to previously unusable spermatozoa from viscous fractions and could improve quality post-thaw. The enzyme papain successfully reduced the viscosity of camelid semen but has yet to be tested in wildlife species. This study assessed the influence of papain on the in vitro quality of rhinoceros spermatozoa during cryopreservation using advanced semen assessment. In experiment 1, the motility of spermatozoa from the viscous fraction of an ejaculate, either untreated or treated with papain and its inhibitor E-64 prior to cryopreservation, was assessed post-thaw. In experiment 2, spermatozoa from papain-treated viscous fractions were compared to spermatozoa frozen from untreated sperm-rich fractions pre-freeze, as well as after 0, 1.5 and 3 h of incubation post-thaw (37 °C). Papain significantly increased the quantity of spermatozoa collected from ejaculates, as well as the motility prior to freezing. Papain also improved the post-thaw motility, velocity, linearity and straightness of samples compared to sperm-rich samples, with no detriment to sperm viability, lipid membrane disorder, production of ROS or DNA integrity (*p* < 0.05). Results show the benefit of supplementing rhinoceros spermatozoa with papain prior to cryopreservation on sperm cryosurvival and demonstrates the potential of using papain to improve the success of cryopreservation protocols, not only for the rhinoceros, but also for other wildlife species.

## 1. Introduction

Current numbers of white, black, greater one-horned, Sumatran and Javan rhinoceros in situ of 10000, 3100, 2200, 30 and 18 adult individuals, respectively [1], emphasise the importance of captive breeding programs for the maintenance of genetic diversity and survival of these species [2,3,4]. Furthermore, it highlights the need for continued development of advanced assisted reproductive technologies (ARTs) to increase the likelihood of success. In this context, efficient collection and cryopreservation of spermatozoa play an important role for artificial breeding programs, in vitro production of embryos and long term genetic biobanking for safeguarding the species survival.

The first successful report of semen cryopreservation in the rhinoceros was published in 1979, reporting a sperm motility of less than 30% post-thaw [5]. Following the adaptation of equine protocols, motilities post-thaw increased to 55% [6,7,8,9], siring the first calf with frozen sperm in 2008 [6]. Since this time, work has continued to try to improve the success of cryopreservation protocols for all rhino species, improving the quality and quantity of spermatozoa post-thaw [10]. Consequently, extensive development of freezing extenders with different cryoprotectants and freezing rates has determined that freezing rhinoceros spermatozoa in ButoCrio^®^ supplemented with glycerol and methylformamide above liquid nitrogen vapour can yield a post-thaw sperm motility of 75.6 ± 3.9% [11]. The presence of seminal plasma has also been investigated during the freezing protocol, with no significant improvements in motility post-thaw noted, if seminal plasma was removed via centrifugation from sperm-rich fractions prior to cryopreservation [11]. Despite these major developments, the use and success of cryopreservation protocols in the rhinoceros is limited by the quality and quantity of the original ejaculate produced [12]. Semen collection in the rhinoceros is predominantly collected via electroejaculation (EJ) [12,13] under anaesthesia [14]. While the development of ultrasonography and species-specific stimulation probes for EJ have resulted in a greater understanding of rhinoceros reproductive anatomy [13,15,16], creating greater consistency in the number of successful collections, the variability in semen quality associated with these collections is often still high [12]. Depending on the duration of accessory sex gland stimulation, the volume and composition of seminal plasma contributing towards the ejaculate can vary [12,13,17]. The ejaculate is collected in 1–6 fractions, with the first 1–2 deemed “sperm-rich” containing the highest concentration of viable sperm. This is followed by subsequent fractions containing large amounts of seminal plasma but lower sperm concentration [12]. In fact, the increasing degree of viscosity in fractions collected after the first, low volume sperm-rich fraction, is a characteristic not only of rhinoceros ejaculates [15,16,17] but also of other wildlife species, including the elephant [18], hippopotamus [19], pangolin [20], primates [21,22] and camelids [23,24]. Even marsupial semen has been reported to coagulate following EJ collection [25,26]. Regardless, the viscosity of ejaculates collected from these species makes the application of ARTs extremely challenging.

High viscosity fractions do not allow for obligatory separation of seminal fluids from spermatozoa by centrifugation or efficient dilution of spermatozoa with cryoprotectants, preventing sperm cryopreservation and ultimately wasting a large proportion of the ejaculate. For example, after successfully collecting 19 ejaculates from 14 African elephant bulls and recording a mean volume of 104.3 ± 80.0 mL and sperm concentration of 781 ± 622.8 × 10^6^ spm/mL, up to 41% was discarded due to the high viscosity associated with fractions [18]. In the white and greater one-horned rhinoceros species, the degree of sperm wastage is even higher. Despite collecting a mean ejaculate volume of 80 ± 15 mL and 158 ± 33 mL, respectively, only 6 ± 1 mL and 26 ± 4 mL of the sperm-rich fractions were cryopreserved, representing a wastage of 92.5% and 84.5% per species [8,11,17]. Given the logistical challenges, potential risk and cost associated with the general anaesthesia of valuable wildlife species, this represents a considerable loss of valuable gametes.

If the viscosity of these collected fractions could be treated and reduced, this could rescue available spermatozoa for ARTs, improving the efficiency of semen collection via electroejaculation. The viscosity of rhinoceros ejaculates has been extensively characterised using a combination of subjective assessment, gel electrophoresis and mass spectrometry techniques [15]. It is mainly caused by secretions of the bulbourethral glands [27], in particular the presence of a 250 kDa glycoprotein, PBU250 [15]. By targeting PBU250 with the enzymes, α-amylase and collagenase, viscosity was reduced by 28% and 21% respectively; however, there was no improvement in the quality of spermatozoa following incubation at room temperature for 1 h [15]. Treatment of camelid semen to reduce viscosity saw greater success [23,28,29,30], recording an improvement in post-thaw sperm quality after the addition of papain, the cysteine protease present in papaya (*Carica papaya*) [30]. When alpaca spermatozoa were treated with 0.1 mg/mL of papain, viscosity was significantly reduced within 30 min, without having any detrimental impact on sperm motility, viability, acrosome integrity and DNA integrity [23]. Furthermore, when samples were frozen, papain-treated spermatozoa exhibited superior total motility post-thaw compared to untreated samples [30,31]. To date, papain has not been examined for its ability to reduce the viscosity of viscous ejaculates in wildlife species, or specifically of rhinoceros ejaculates, and holds tremendous promise to improve cryopreservation protocols in the rhinoceros as well as other endangered wildlife species with viscous ejaculates.

If papain was found to be successful, the yield of viable spermatozoa produced from one semen collection would significantly increase that which is available for use in advanced ARTs. It would also allow the use of advanced semen assessment tools, such as fluorescent flow cytometry, to examine more advanced sperm-cell characteristics, currently applied to spermatozoa from other domestic species. This additional information would ultimately contribute towards a better understanding of the fertility status of preserved rhinoceros spermatozoa, aiding in the success of ARTs. Furthermore, using the southern white rhinoceros as a model, papain may be a viable tool to reduce the viscosity and increase the yield of spermatozoa collected in other rhinoceros species, or from other wildlife species, such as elephants, tapirs, hippopotamus and primates, where electroejaculation is the method of choice for semen collection.

As such, the following study will aim to: (1) examine the influence of papain on the post-thaw quality of spermatozoa frozen from viscous or low sperm ejaculate fractions, (2) compare the post-thaw quality of papain-treated spermatozoa to that of sperm-rich samples, and (3) objectively assess, for the first time, advanced membrane characteristics of post-thaw rhinoceros spermatozoa using flow cytometry. It was hypothesised that the benefit of papain would be two-fold. It would increase the quantity of rhinoceros spermatozoa available for ARTs post collection, as well as improve the quality of spermatozoa post thaw.

## 2. Materials and Methods

### 2.1. Chemicals and Reagents

Unless otherwise stated, all chemicals were supplied by Sigma-Aldrich (St. Louis, MO, USA).

Low sperm, viscous fractions were diluted in a modified Tyrodes solution (TALP), which consisted of 2 mM CaCL_2_, 3.1 mM KCl, 0.4 mM MgCL_2_, 95 mM NaCl, 0.3 mM NaH_2_PO_4_, 10 mM HEPES, 21.6 mM Na Lactate, 5 mM glucose, 1 mM Na pyruvate, 25 mM NaHCO_3_, 0.03 mM phenol red and 3 mg/mL BSA (fatty acid free).

### 2.2. Ethics Statement

This study was conducted on captive southern white rhinoceros (*Ceratotherium simum*), a species listed on the IUCN Red List of Threatened species™ as Near Threatened [32]. Semen was collected from bulls as part of a clinical examination of their fertility or for use in artificial inseminations. The study was approved by the IACUC animal ethics committee of the Leibniz Institute for Zoo and Wildlife Research (permit number: 2017-08-02).

### 2.3. Experimental Design

Two experiments were conducted to assess the effectiveness of the enzyme papain on improving the quality of spermatozoa sourced from low sperm fractions. Experiment 1 was a proof-of-concept study, where half of the viscous or low sperm fractions of each ejaculate collected (n = 4) was treated with papain (Sigma, St. Louis, MO, USA) before being processed for cryopreservation. Sperm motility was immediately assessed post-thaw on a computer-assisted sperm analyser (CASA; Hamilton Thorne IVOS II and CEROS II, Animal Breeder software, Version 1.8; Beverly, CA, USA) and compared to the non-treated viscous sample. Experiment 2 treated the low sperm, viscous ejaculate fractions collected (n = 6) with papain prior to cryopreservation. Sperm quality post thaw was then compared to spermatozoa frozen from the sperm-rich fraction of the same bull, immediately after thawing, as well as 1.5 and 3 h after incubation (37 °C). Spermatozoa were assessed for motility and associated kinematics using CASA. Membrane viability, acrosome integrity, lipid membrane disorder, production of reactive oxygen species and DNA integrity were also assessed using flow cytometry (Cytoflex; Beckman Coulter, Lane Cove, Australia and C6 BD Accuri; Becton Dickinson, NJ, USA).

### 2.4. Animals and Semen Collection

Over a 4-year period, semen was collected via electroejaculation (Seager model 14, Dalzell USA Medical Systems, The Plains, VA, USA) from 10 adult, captive, white rhinoceros bulls (n = 10) located at 3 European (Exp 1; n = 4 bulls) and 4 Australian (Exp 2; n = 6 bulls) zoos. For semen collection by electrostimulation, general anaesthesia in lateral recumbency was required. For this, 25 mg detomidine hydrochloride (Domidine1 10 mg/mL, Eurovet Animal Health B.V., Bladel, The Netherlands) and 25 mg butorphanol (Torbugesic1 Vet 10 mg/mL, Zoetis B.V., Capelle a/d IJssel, The Netherlands) were injected intra-muscularly as a premedication. A combination of 150 mg ketamine hydrochloride (Ketamin 10% WDT, Henry Schein VET GmbH, Hamburg, Germany) and 1.8–2.7 mg etorphine (Captivon, Wildlife Pharmaceuticals South Africa, Karino, South Africa) was injected intravenous into the ear vein after 20 min. Anaesthesia was antagonised by administration of 250 mg naltrexone hydrochloride (TrexonilTM, Wildlife Pharmaceuticals (PTY) Ltd., White River, South Africa) and 40 mg atipamezole hydrochloride (Atipam 5 mg/mL, Eurovet Animal Health B.V.). Half the reversal was given i.m. and the other half was given i.v. Animals were normal and alert two to three minutes after the antagonist was given. For electroejaculation, a custom-made electric probe, specifically designed for rhinoceros, was used for stimulation [27]. The stimulation probe expanded the lumen of the rectum providing maximum electric coupling of electrodes. Multiple sets of 2–3 electrical stimuli were applied with increasing voltage and amperage (5–15 V/200–800 mA). Each set of stimulations was followed by manual massage of the pelvic and penile aspects of the urethra [7,17]. After each set of stimulations, up to 6 fractions were collected into foam-insulated 50 mL isotherm collection tubes, labelled and transferred to the onsite laboratory at body temperature, where they were immediately analysed.

### 2.5. Assessment of Initial Ejaculate and Sperm Membrane Characteristics

Each fraction collected per bull was assessed for volume, concentration and total sperm produced per fraction. Sperm concentration was determined using a haemocytometer, as described by Evans and Maxwell [33], and total sperm per fraction (×10^9^) was calculated by multiplying fraction volume by concentration (×10^6^ sperm/mL).

A small aliquot of each fraction was assessed for motility approximately 20 min after collection. The percentage of motile spermatozoa was estimated subjectively to the nearest 5% on heated slides (37 °C) after examining several fields of view on a phase contrast light microscope (Olympus C41, Olympus, Germany). In experiment 2, only sperm-rich fractions which recorded a motility score of 40% and above were deemed acceptable for cryopreservation (as per current standards) and were included in the study.

Assessment of sperm membrane characteristics were conducted as described below. Acrosome integrity and morphology were assessed by fixing 10 μL aliquots of sample in 40 μL of Hancocks fixative [34]. Acrosomes were classified as intact versus modified or reacted (including completely detached acrosomes). Sperm morphology was assessed by visually searching several fields of view for a wide range of abnormalities. Assessment of sperm viability prior to freezing was evaluated using a hypo-osmotic swelling test (HOS), as described by Reid, Hermes, Blottner, Goeritz, Wibbelt, Walzer, Bryant, Portas, Streich and Hildebrandt [9]. Briefly, an aliquot of 20 µL of each sample was diluted in 100 µL hypo-osmolar TALP (100 mOsm), incubated at 37 °C for 30 min and subsequently fixed with paraformaldehyde (18.5%, 100 µL). From each sample, 10 µL was prepared on a glass slide with coverslip, whereby 200 sperm were evaluated. Spermatozoa were categorised as HOS-negative or non-viable when they remained unchanged in the hypo-osmotic environment. Swollen sperm or sperm with curved tails were classified as HOS-positive or viable spermatozoa with functional membranes.

### 2.6. Treatment of Viscous Fractions with Papain and E-64

Prior to enzyme treatment, viscous fractions were diluted 1:1 with a modified Tyrode’s medium warmed to 37 °C and supplemented with albumin, lactate and pyruvate (TALP) [35], and 3 mg/mL BSA was added fresh on the day of collection.

Half of the sample (Exp 1) or all of the sample (Exp 2) was then incubated with papain (0.1 mg/mL; Sigma 76216, St. Louis, MO, USA) at 37 °C for 30 min, (Papain-treated; Exp 1 and 2). In experiment 1 the remaining sample was left untreated (Viscous control; Exp 1) before being processed for cryopreservation.

Following treatment, the reaction was then suspended with the addition of an inhibitor, trans-epoxysuccinyl-L-leucylamido (4-guanidino)butane (E-64;10 μM; Sigma E3132, St. Louis, MO, USA) and incubated at 37 °C for 5 min [30].

To demonstrate the change in viscosity due to papain treatment, the time taken for aliquots (3 μL) of treated and non-treated viscous fractions to fill a standard 20 µm Leja CASA slide (Hamilton Thorne, Beverly, MA, USA) was recorded (n = 1 bull only), as previously described [36].

### 2.7. Dilution and Cryopreservation of Rhinoceros Spermatozoa

Prior to cryopreservation, all samples (Exp 1: papain-treated and viscous control treatments; Exp 2: papain-treated and sperm-rich treatments) were centrifuged (1000× *g*; 20 min; room temp), underlain with 1 mL of a high-density gradient (OptiPrepTM, Sigma–Aldrich, Taufkirchen, Germany) to remove enzymes, seminal plasma and concentrate spermatozoa. Following centrifugation, the supernatant was discarded, the sperm-rich layer above the density gradient aspirated and concentration determined using a haemocytometer.

Samples were then resuspended in ButoCrio^®^ (Nida-con, Mölndal, Sweden) to an approximate concentration of 100–150 × 10^6^ sperm/mL.

Sperm suspensions were loaded into 0.5 mL straws and chilled for 45 min to 4 °C (0.5 °C/min). Straws were then held 4 cm above liquid nitrogen vapour for 8 min, before being plunged into liquid nitrogen. Straws were stored in liquid nitrogen until assessment in the laboratory.

### 2.8. Thawing and Advanced In Vitro Semen Assessment

Two straws per sample were thawed in a 37 °C water bath for approximately 60 s with gentle agitation and incubated over 3 h (Exp 2 only). Aliquots were then taken at 0, 1.5 and 3 h and diluted to 20 × 10^6^ spermatozoa/mL with TALP supplemented with 3 mg/mL BSA. Spermatozoa was assessed for motility and other kinematics via computer-assisted sperm analysis (CASA CEROSII (Exp 1) IVOS II (Exp 2); Hamilton Thorne, USA). Viability, acrosome integrity, production of reactive oxygen species, membrane fluidity and DNA integrity of spermatozoa (Exp 2 only) was performed using fluorescent staining and flow cytometry (Cytoflex; Beckman Coulter, Lane Cove, AUS; C6 BD Accuri, Becton Dickinson, NJ, USA) following consideration of the recommendations published by Lee, et al. [37].

#### 2.8.1. Computer-Assisted Sperm Analysis (CASA)

Post-thaw motility was assessed using CASA. Semen samples (5.5 µL) were placed on prewarmed slides (Cell Vu, Millenium Sciences Corp., New York, NY, USA) immediately prior to assessment. Motility (TM) and other kinematic parameters, including progressive motility (PM), average path velocity (VAP), straight line velocity (VSL), curvilinear velocity (VCL), amplitude of lateral head displacement (ALH), beat cross frequency (BCF), straightness (STR) and linearity (LIN) of spermatozoa was determined by capturing 8 microscopic fields (recording ≥ 200 cells/sample) using the following settings established for rhinoceros: frame rate = 60 Hz; number of frames acquired = 60; minimum contrast = 65; head size minimum 10 µm^2^; head size maximum 30 µm^2^; medium average path velocity cutoff = 25 µm/s; medium threshold straightness = 75%; slow average path velocity cutoff = 20 µm/s; slow straight line velocity cut off = 6 µm/s; head brightness minimum = 157; tail brightness minimum = 75; magnification = 1.2; illumination intensity = 85; temperature = 37 °C.

#### 2.8.2. Flow Cytometric Analysis

Flow cytometric analysis of viability, acrosome integrity, reactive oxygen species and lipid membrane disorder was performed using a Cytoflex flow cytometer calibrated prior to use with Cytoflex daily QC fluorospheres (Beckman Coulter, Lane Cove, Australia). Sperm cells were isolated from total events based on forward and side scatter profiles using 3 different lasers including 50 mW 488 nm, 50 mW 638 nm and 80 mW 405 nm. Further separation from debris was performed by staining samples with Hoechst 33342 at a final concentration of 1 ug/mL. For every tested parameter, 10,000 cells were analysed per sample using CytExpert 2.0 software (Beckman Coulter, Lane Cove Australia).

The viability and acrosome integrity of sperm cells was assessed using a combination of fluorescein isothiocyanate (FITC)-PNA (final concentration; 0.4 μg/mL) and propidium iodide (PI, final concentration; 6 μM). Samples were incubated for 10 min at 37 °C prior to assessment. Propidium iodide fluorescence was detected using a 610/20 band-pass filter, and FITC-PNA fluorescence was detected on a 525/40 nm band-pass filter. If sperm cells were classified as both FITC-PNA and PI negative, they were considered viable cells with intact acrosomes.

The proportion of viable cells detected with lipid membrane disorder was assessed using a combination of merocyanine 540 (M540, final concentration; 0.8 μM) and YO-PRO (final concentration; 25 nM). Samples were incubated for 10 min at 37 °C prior to assessment. M540 fluorescence was detected on a 585/40 nm band-pass filter and Yo-Pro fluorescence detected on a 525/40 nm band-pass filter. Cells which were classified as Yo-Pro-negative were viable, with higher median M540 fluorescence corresponding to high levels of lipid disorder and membrane fluidity.

The proportion of viable cells with high levels of intracellular reactive oxygen species (ROS) was assessed using a combination of dichlorodihydrofluorescein diacetate acetyl ester (H2DCFDA, final concentration; 5 μM) and PI (final concentration; 6 μM). Samples were incubated with H2DCFDA for 60 min at 37 °C before being centrifuged (10 min at 600× *g*), supernatant removed, and sperm pellets resuspended in fresh buffer (TALP +0.3% BSA). An aliquot of this new sperm suspension was then counterstained with PI for 10 min at 37 °C at each time point for assessment. H2DCFDA fluorescence was detected on a 525/40 band-pass filter. Cells which were PI negative were classified as viable with higher median H2DCFDA fluorescence levels corresponding to a high production of intracellular ROS.

Flow cytometric analysis of the DNA integrity of frozen-thawed rhinoceros spermatozoa was performed using a C6 BD Accuri (Becton Dickinson, NJ, USA). Samples were stained with acridine orange (final concentration; 6 μg/mL) as described by Pool, et al. [38]. Stained samples were incubated for 3 min before assessment, where green fluorescence (FL1) was detected using a 533/30 band-pass filter, and red fluorescence (FL3) was detected using a 670 long pass filter. Flow rate was set to around 200 events per second, and a minimum of 5000 sperm cells were recorded per sample. DNA fragmentation was estimated by the relative amount of single-stranded and double-stranded DNA, indicated by the proportion of sperm demonstrating red fluorescence to total fluorescence or cells outside the main population.

### 2.9. Statistical Analysis

Initial ejaculate characteristics, motility (Exp 1), viability, acrosome integrity and percent normal morphology (Exp 2 only) assessed prior to freezing, were analysed using an ANOVA and means were compared using a two-sample t-test comparison of means assuming unequal variances. Post-thaw motility kinematics (Exp 1) were assessed using an ANOVA and means were compared using a paired two-sample *t*-test. *t*-tests were conducted in Genstat (Version 18, VSN International, Hemel Hempstead, UK).

In Experiment 2, sperm motility, viability, acrosome integrity, lipid membrane disorder, intracellular ROS production and DNA integrity were analysed using a restricted maximum likelihood model (REML) in Genstat. Treatment and timepoint (0, 1.5 or 3) were specified as fixed effects, while individual bull was specified as a random effect. REMLS were used in the current study to investigate whether there was any interaction between the influence of treatments on changes in sperm parameters over time. These interactions were dropped from the model if not significant (*p* > 0.05).

Normal distribution of data and homogeneity of variances were assessed within Genstat. For both experiments and variables, means were reported with ± S.E.M and *p* < 0.05 was considered statistically significant.

## 3. Results

### 3.1. Initial Ejaculate Characteristics Collected in Experiment 1 and 2 Prior to Freezing

**Figure 1:**
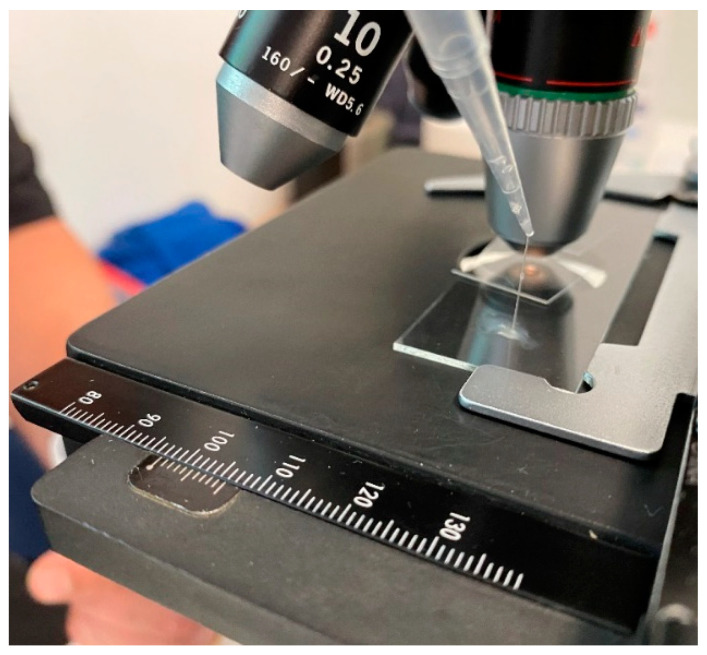
Visual example of viscosity witnessed in non-sperm-rich fraction of rhino ejaculate collected via electro-ejaculation.

Table 1 presents the average number of ejaculates collected, number of ejaculates utilised, average volume, concentration and total sperm number of both the sperm-rich and viscous, low sperm fractions collected via electroejaculation from white rhinoceros bulls (n = 10). Across both experiment 1 and 2, there was a total of 11 ejaculates collected from 9 bulls over the collection period. Of these 11 ejaculates, only 10 contained a sperm-rich fraction deemed acceptable for further use in the study. Figure 1 provides a visual example of the viscosity noted in viscous, low sperm fractions during the study. Further, when a standard 20 µm Leja CASA slide was loaded with 3 µL of the viscous sperm fraction, it took 17 s for the chamber to load. Aliquots of papain-treated fractions filled the 20 µm chamber within 5 sec (n = 1 bull). There was a significant difference in the volume and concentration of spermatozoa (spermatozoa/mL) collected between the sperm-rich and viscous, low sperm fractions. The viscous, low sperm fraction recorded a larger volume but smaller sperm concentration compared to the sperm-rich fraction (*p* < 0.05; Table 1). There was no significant difference in the total number of sperm collected in both fractions (×10^9^ spermatozoa; *p* > 0.05; Table 1). 

### 3.2. Papain Improves the Quality of Spermatozoa Originating from Viscous, Low Sperm Fractions Both Prior to and after Cryopreservation

Table 2 presents the characteristics of samples collected and assessed in Experiment 1, prior to freezing as well as immediately post-thaw. Papain-treated samples recorded a significantly higher subjective motility prior to freezing compared to non-treated samples (88.3% ± 1.70% vs. 78.8% ± 2.39%, respectively; *p* < 0.05; Table 2). Post-thaw, papain-treated samples also recorded a significantly higher TM compared to untreated samples (84.9% ± 1.60% vs. 37.5% ± 10.56%, respectively; *p* < 0.05; Table 2), with almost no difference in the pre-freeze and post-thaw motility of treated samples.

Post-thaw, the PM, VAP, VCL, VSL and ALH of papain-treated samples in experiment 1 was significantly higher compared to the non-treated viscous samples immediately post-thaw (*p* < 0.05; Table 2). There was no significant difference in the post-thaw BCF, LIN or STR of treated and non-treated spermatozoa originating from the viscous fraction (*p* > 0.05; Table 2).

The viability of viscous spermatozoa was significantly higher post-thaw if treated with papain prior to freezing, compared to the untreated control (86.3 ± 3.33% vs. 71.5 ± 3.66, respectively; *p* < 0.05).

Table 3 displays the pre-freeze and post-thaw sperm characteristics of samples collected and analysed in Experiment 2, where viscous spermatozoa treated with papain were compared to spermatozoa from the sperm-rich fraction. There was no significant difference in the mean sperm motility of treatments prior to freezing (*p* > 0.05; Table 3).

Post-thaw, there was no significant interaction of treatment and time on the TM of frozen-thawed rhinoceros spermatozoa. In other words, there was no significant difference in the decline of treatments over time. However, when pooled over time, spermatozoa treated with papain prior to cryopreservation recorded a significantly higher motility than the sperm-rich control (*p* < 0.05; Table 3). When pooled over treatment, the TM of spermatozoa post-thaw declined significantly over time from 69.3% ± 3.10% at 0 h, 44.0% ± 6.91% to 1.5 h and 24.4% ± 4.37% at 3 h (*p* < 0.001).

There was no significant interaction of treatment and time on the PM of spermatozoa. However, when pooled over time, spermatozoa treated with papain recorded significantly higher PM compared to the sperm-rich sample (*p* < 0.05; Table 3). When pooled over treatment, the PM of spermatozoa post-thaw declined significantly over time from 45.8% ± 4.67% at 0 h to 32.9% ± 6.87% at 1.5 h and 15.6% ± 3.63% at 3 h (*p* < 0.001).

There was no significant interaction of treatment and time on the VAP, VCL or VSL of spermatozoa. However, when pooled over time, the VAP, VCL and VSL of spermatozoa treated with papain prior to cryopreservation was significantly higher compared to the sperm-rich control samples (*p* < 0.05; Table 3). When pooled over treatment, the VAP, VCL and VSL of spermatozoa post-thaw declined significantly over time from 75.1 ± 6.57, 118.0 ± 11.76 and 58.4 ± 4.67 µm/s at 0 h to 59.0 ± 6.58, 86.2 ± 8.47 and 51.0 ± 6.53 µm/s at 1.5 h and finally 42.0 ± 4.83, 64.5 ± 6.91 and 58.4 ± 4.67 µm/s at 3 h (*p* < 0.001).

There was no significant interaction of treatment and time on the STR or LIN of frozen-thawed rhinoceros spermatozoa. However, when pooled over time, the STR and LIN of papain-treated spermatozoa was significantly higher compared to the sperm-rich control samples (*p* < 0.05; Table 3). When pooled over treatment, there was a significant effect of time on the LIN but not the STR of spermatozoa post-thaw. The LIN of samples increased from 0 h (54.6% ± 3.03%) to 1.5 h (61.7% ± 2.18%; *p* < 0.05). There was no difference between 1.5 and 3 h (60.1% ± 3.08%; *p* > 0.05).

There was no significant interaction of treatment and time on the BCF or ALH of spermatozoa post-thaw. However, when pooled over treatments, there was a significant effect of time on the BCF and ALH of spermatozoa post-thaw. The BCF of spermatozoa was significantly lower at 3 h (36.6 ± 1.50%) post-thaw compared to 0 h (41.0% ± 0.96%) and 1.5 h (41.0% ± 0.96%; *p* < 0.05). The ALH of spermatozoa was significantly higher at 0 h (4.5% ± 0.36%) compared to 1.5 h (3.3% ± 0.23%) and 3 h (2.9% ± 0.31%; *p* < 0.001). There was no effect of treatment alone on the BCF and ALH of spermatozoa (*p* > 0.05).

### 3.3. Papain Does Not Influence the Viability, Acrosome Integrity, Membrane Lipid Disorder, Intracellular ROS Production or DNA Integrity of Frozen-Thawed Rhinoceros Spermatozoa Assessed Using Flow Cytometry

In experiment 2, prior to freezing, there was no significant difference between the percent viable, acrosome intact or normal morphology of sperm-rich and papain-treated samples (*p* > 0.05; Table 3). There was no significant interaction of time and treatment on the percentage of viable, acrosome intact spermatozoa post-thaw. Nor was there any significant effect of treatment when pooled over time (*p* > 0.05; Table 3). However, when pooled over treatments, the percentage of viable acrosome-intact spermatozoa was significantly lower at 3 h (30.1% ± 2.66%) compared to 0 h (48.4% ± 1.94%) and 1.5 h (41.3% ± 2.91%; *p* < 0.001).

There was no significant interaction of time and treatment on the median level of H2DCFDA or M540 fluorescence, otherwise known as level of ROS production and membrane lipid disorder, respectively (*p* > 0.05; Table 3). Nor was there any significant effect of treatment or time alone (*p* > 0.05).

There was no significant interaction of time and treatment on the percentage of DNA fragmentation (*p* > 0.05; Table 3). Nor was there any significant effect of treatment or time alone (*p* > 0.05).

## 4. Discussion

The near-threatened southern white rhinoceros serves as an excellent example of a species whose semen is characteristically viscous post-semen collection. This is caused in part by the presence of particular components within the seminal plasma, secreted in large amounts when the accessory sex glands are stimulated during electro-ejaculation [12,15]. This phenomenon is shared by other species, including camelids [23,28], elephants [18,34], hippopotamus’ [19] and primates [21,22]. Spermatozoa trapped within the viscous (low sperm) fractions of the ejaculate equate to almost half of the total spermatozoa collected [11,17,20], but are often discarded. In the current study, treatment of viscous, low sperm rhinoceros spermatozoa, with papain prior to cryopreservation, not only enabled the utilisation of spermatozoa within these fractions, but resulted in improved motility and viability post-thaw. It also resulted in a similar motility to sperm-rich spermatozoa prior to freezing, however, it surprisingly exceeded the motility of sperm-rich treatments post-thaw. Furthermore, papain was not detrimental to the membrane or acrosome status of spermatozoa, nor found to have any impact on the level of intracellular reactive oxygen species (ROS) produced, lipid membrane disorder or degree of DNA fragmentation when compared to sperm-rich treatments, key characteristics for the timely maturation and survival of spermatozoa within the female. As such, treatment of viscous, low sperm rhinoceros fractions with papain increased sperm yield by almost two-fold post collection. Considering the ethical, logistical and financial challenges associated with the collection of semen from threatened or endangered wildlife species, this represents a substantial improvement in the efficiency of existing semen collection and cryopreservation protocols for the rhinoceros and other species which suffer from viscous ejaculates post collection.

Treatment of rhinoceros spermatozoa with papain enabled the assessment and successful cryopreservation of rhinoceros spermatozoa from the viscous, low sperm fraction of the ejaculate. In the current study, despite the sperm-rich fractions recording higher sperm concentration per mL compared to the viscous fractions (as shown previously [12,17]), given the larger volume produced, the total number of spermatozoa contained within this fraction (3.5 ± 0.79 × 10^9^ sperm) was similar to that produced by the sperm-rich fraction (2.0 ± 0.98 × 10^9^ sperm), spermatozoa which would have been discarded, if not for the addition of papain. Consequently, this enabled the cryopreservation of an additional 3.5 billion cells on average per male. To the best of our knowledge, when both fractions are combined, this amount exceeds what has previously been reported (2.8 ± 0.8 × 10^9^) for high quality spermatozoa collected from the southern white rhinoceros via electroejaculation [10,17]. It is also higher than that recorded for the black (200 × 10^6^ spm [13]) and Sumatran (2.5 × 10^9^ [39]) rhinoceros. Although the greater one-horned rhinoceros recorded an impressive yield of 30.4 × 10^9^ spm [8], the quality associated with this collection was not confirmed. The importance of improving the quantity of gamete cryopreservation in endangered species is best demonstrated in the Sumatran rhinoceros, where semen collection attempts have returned poor results. With the species listed as critically endangered, successful semen collection attempts are paramount. This would enable the rapid generation of a biobank containing genetically distinct gametes to help safeguard the species from extinction—a luxury not afforded to the northern white rhinoceros species, where in vitro fertilisation of oocytes harvested from the one last remaining female, depends on the quantity and quality of frozen spermatozoa collected from one deceased male.

The treatment of viscous fractions with papain not only resulted in an increase in sperm quantity collected per ejaculate, it also demonstrated the ability to improve motility prior to and following freezing. Following papain treatment and dilution in Butocrio, the motility of spermatozoa from viscous, low sperm fractions prior to freezing was improved by 10%. Post-thaw, papain-treated samples displayed superior freezing resilience (motility and viability) compared to untreated viscous samples, as well as samples frozen from the sperm-rich fraction. The post-thaw motility of the sperm-rich samples recorded in the current study was similar to that recorded previously in southern white rhinos by O’Brien, et al. [40] but lower than that reported by Hermes, Hildebrandt and Göritz [11]. We hypothesise this difference to be related to the age, sexual maturity and original semen quality of several bulls included in Exp 2. In any event, the low post-thaw motility of the sperm-rich samples in the current study highlights more so the improvements which could be made following treatment with papain, evidenced by a 16% increase in post-thaw motility when compared to the sperm-rich control (Table 2). This could also suggest a beneficial effect of papain on spermatozoa originating from the sperm-rich fraction, where removal or enzymatic treatment of any existing seminal plasma prior to freezing could improve survival rates. While seminal plasma has been shown to be beneficial to the freezing process for species such as the ram [41], its presence during stallion sperm cryopreservation has also been questioned [42,43]. This similarity in the contradictory nature of seminal plasma during freezing is unsurprising given that the horse is the closest domestic relative of the rhinoceros [44]. Regardless, the above results suggest that treatment of all rhinoceros ejaculate fractions with papain could be beneficial for improving the cryosurvival of spermatozoa for ARTs.

The mode of action by which papain improves the quality of rhinoceros semen collected from viscous fractions remains to be fully understood. We suspect that the issue of semen viscosity is not related to the volume of plasma produced but rather the presence or concentration of particular protein/s. We also know from previous studies in alpaca, that the proteinase papain targets glycosylated proteins within the seminal fluid, rather than glycosaminoglans [23]. This strongly supports the findings by Behr and colleagues in 2009, who identified the presence of a 250 kDa glycoprotein (P250) within the viscous seminal plasma of southern white rhino seminal plasma originating from the bulbourethral gland [15]. The occurrence and intensity of P250 also correlated strongly with increasing grades of fraction viscosity [15]. In other species, glycoproteins secreted by the bulbourethral glands have contributed to both the gelatinous plug produced at the end of the boars ejaculate [45,46] and the entrapment of spermatozoa within the uterus of camelids [47]. With this in mind, it is highly likely that in the current study, papain reduced the viscosity of ejaculates by targeting P250. This breakdown of P250 allowed spermatozoa to swim more freely after dilution and increased the uniformity of cryoprotectant exposure, ultimately contributing to better protection during the freezing process. This, in turn, saw a benefit for the survival of spermatozoa from the viscous fraction post-thaw, particularly in relation to the noted improvement in cell viability.

Of course, the identity of this protein is the missing part of the puzzle and would help enable future research to confirm our hypothesis. At the time of publication, although good mass spectrometry spectra was generated, Behr and colleagues, and colleagues in 2009 [15] failed to match a protein identity to P250. However, since this time significant development of the rhinoceros genome has occurred, so repeating the search against the genome would be prudent to determine any further leads. It would also be interesting to see if this protein is conserved across other species of rhinoceros, such as the critically endangered black [48], Sumatran [49] and Javan [50] rhinoceroses and vulnerable greater one-horned rhinoceros [51], particularly the Sumatran rhinoceros, given less than 30 individuals remain, and all published reports of semen collection report severely low sperm counts and volume [39,52]. Similarly, comparison against the seminal plasma proteome of the hippopotamus [19], elephants [20,35] and primates [22] would show whether this protein is responsible for causing the production of viscous fractions across a wide variety of species. More so, it would confirm whether papain could be used as a tool to improve the quantity and quality of spermatozoa collected and cryopreserved from wildlife species following electro-ejaculation.

Importantly, when compared to spermatozoa from the sperm-rich fraction, the viability, acrosome integrity, production of reactive oxygen species and lipid membrane disorder was not impacted following treatment with papain. In other words, papain was not detrimental to the functioning of the sperm cell. Similar results were seen in the alpaca where viscosity was reduced with papain [23], and rhino with α-amylase and collagenase [15]; however, no improvement in the ability of rhinoceros spermatozoa to survive freezing was seen either. Sperm membrane characteristics were likely preserved by the addition of the inhibitor, E-64, which prevented any prolonged exposure and eventual target or degradation of the sperm cell during the chilling and freezing process [30]. E-64 is a cysteine protease inhibitor which binds to the active thiol group and reduces the functionality of papain [53,54]. The concentration of papain and E-64 used in the current study mirrored that reported in the alpaca studies [30]. This concentration (0.1 μg/mL) was successful at reducing the viscosity of alpaca semen, with no detriment to freezing or pregnancy rates following AI of females [31]. Pleasingly, 0.1 μg/mL was also successful at reducing the viscosity of rhinoceros samples completely. However, it could be useful to further optimise the protocol in case further improvements could be made, such as the length of enzyme incubation, to reduce overall processing time. Confirmation of pregnancy following AI in the rhinoceros would also be beneficial.

Furthermore, to the best of our knowledge, the current study reports, for the first time, the advanced membrane and molecular characteristics of white rhinoceros spermatozoa, assessed post-thaw using flow cytometry. The viability reported post-thaw is similar to the 48% reported in 2009 [6] which assessed the percentage of live cells using a hypo-osmotic swelling (HOS) test prior to artificial insemination. Lipid membrane disorder or membrane fluidity, as measured by median M540 fluorescence, is considered an early indication of capacitation and apoptotic-like changes [55], a sperm maturation step which occurs within the female and is crucial for fertilisation (for a review please see [56]). Premature capacitation of spermatozoa is a common artifact of freezing and can lead to early apoptosis of the cell [57]. Production of intracellular reactive oxygen species (ROS), as measured by median H2DCFDA fluorescence, is a measure of the harmful free radicals produced during sperm metabolism. If left uncontrolled, excessive ROS levels can lead to cell degradation and DNA damage [58]. Further examination of these rhinoceros sperm cell characteristics is vital to enhance understanding of the molecular nature of rhinoceros spermatozoa during the freezing process, and of how these may influence the outcome of artificial insemination programs, for which the rhinoceros currently records a low success rate [3,4]. Nevertheless, the above results provide an important benchmark for the species.

## 5. Conclusions

Assisted reproductive technologies are becoming increasingly diverse, complex and revolutionary in their development and application to wildlife species. Cryopreservation and biobanking of gametes, tissues and cell lines from endangered species has the potential to preserve gametes for the long term, maintain genetic diversity and help fight against the threat of extinction. For example, the successful collection of male gametes from the endangered Sumatran rhinoceros in terms of quantity and quality can play a key role in the de-extinction of the species in the future [4]. The current study has contributed towards this goal by demonstrating the ability of papain to ensure high quantities of viable rhinoceros spermatozoa are collected from ejaculates, even if fractions are deemed low sperm and viscous. It has also proven its ability to increase sperm motility and velocity post-thaw, with no detriment to membrane fluidity, acrosome integrity or intracellular ROS levels. In combination, this data identifies papain as a viable tool to improve the success, not only of rhinoceros cryopreservation protocols, but potentially of other wildlife species, where ARTs are limited by viscous ejaculates, the outcomes of which could contribute towards more successful ARTs and the survival of threatened or endangered species for years to come.

## Figures and Tables

**Table 1:** Semen characteristics of the sperm-rich and viscous, low sperm fractions collected in Experiment 1 and 2, from southern white rhinoceros bulls (n = 9) via electroejaculation. Data is mean ± SEM. Columns which differ in superscript indicate significant differences between means (*p* < 0.05).

Parameter	Ejaculate Fraction	
	Sperm-Rich	Viscous, Low Sperm
Number of ejaculate fractions collected	10	11
Volume (ml)	2.6 ± 0.58 ^a^	15.1 ± 2.4 ^b^
Concentration (×10^6^ spermatozoa/mL)	863.3 ± 116.99 ^a^	236.0 ± 46.61 ^b^
Total sperm number per ejaculate (×10^9^ spermatozoa)	2.7 ± 0.93	3.4 ± 0.71

**Table 2:** Sperm characteristics of viscous, low sperm control and papain-treated samples collected from southern white rhinoceros bulls (n = 3 bulls, 4 ejaculates) assessed prior to and following cryopreservation (Exp 1). Percent viability, acrosome intact and normal morphology was assessed subjectively. Data are mean ± SEM. Columns which differ in superscript indicate significant differences between the viscous control and viscous treated samples (*p* < 0.05).

Time	Sperm Parameters	Viscous, Low Sperm Fraction	
		Non-Treated Control	Papain-Treated
Pre-freeze	Subjective motility (%)	78.8 ± 2.39 ^a^	88.3 ± 1.7 ^b^
Post-thaw	TM (%)	37.5 ± 10.56 ^a^	84.9 ± 1.60 ^b^
	PM (%)	25.4 ± 9.32 ^a^	56.6 ± 5.72 ^b^
	VAP	43.8 ± 1.92 ^a^	74.4 ± 5.48 ^b^
	VCL	77.1 ± 3.16 ^a^	115.8 ± 8.30 ^b^
	VSL	34.7 ± 4.58 ^a^	57.6 ± 3.51 ^b^
	ALH	3.7 ± 0.40 ^a^	4.5 ± 0.18 ^b^
	BCF	39.6 ± 2.81	41.2 ± 0.23
	LIN	50.8 ± 6.02	52.0 ± 1.86
	STR	80.9 ± 5.92	78.3 ± 2.87
	Percent viable (%) Percent acrosome intact (%) Percent normal morphology (%)	71.5 ± 3.66 ^a^	86.3 ± 3.33 ^b^
		68 ± 6.48	80.0 ± 3.70
		70.8 ± 4.37	80.5 ± 2.72

**Table 3:** Pre-freeze and post-thaw characteristics of sperm-rich and viscous low sperm papain-treated spermatozoa collected from southern white rhinoceros bulls (n = 6) following treatment, centrifugation and dilution in ButoCrio (Exp 2). Data is pooled over bull and time (0, 1.5 and 3 h post-thaw) ± SEM. Columns which differ in superscript indicate significant differences between sperm-rich control and papain-treated spermatozoa (*p* < 0.05).

Time Point	In Vitro Sperm Parameters	Sperm-Rich Control	Viscous Low Sperm Papain-Treated
Pre-freeze	Subjective motility (%)	78.4 ± 6.66	76.6 ± 5.22
	Percent viable (%)	92.0 ± 1.10	93.2 ± 0.92
	Percent acrosome intact (%)	88.2 ± 2.48	89.2 ± 1.86
	Percent normal morphology (%)	82.2 ± 4.59	78.0 ± 5.10
Post-thaw	TM (%)	38.0 ± 5.50 ^a^	54.2 ± 6.13 ^b^
	PM (%)	21.8 ± 3.99 ^a^	40.9 ± 5.20 ^b^
	VAP (µm/s)	48.3 ± 4.41 ^a^	68.8 ± 6.04 ^b^
	VCL (µm/s)	78.0 ± 8.19 ^a^	101.2 ± 9.45 ^b^
	VSL (µm/s)	37.9 ± 3.38 ^a^	58.9 ± 4.60 ^b^
	LIN (%)	9.0 ± 2.33 ^a^	8.15 ± 1.98 ^b^
	STR (%)	81.0 ± 2.04 ^a^	87.4 ± 1.79 ^b^
	BCF (%)	39.0 ± 1.30	40.2 ± 0.96
	ALH (%)	3.66 ± 0.31	3.57 ± 0.29
	Viable acrosome intact (%)	40.2 ± 3.89	39.3 ± 1.90
	Membrane lipid disorder (median M540 fluor.)	2966.4 ± 357.26	13,993.6 ± 4901.47
	Level of ROS (median H2DCFDA fluor.)	17,278.9 ± 3923.57	9073.0 ± 2103.47
	DNA integrity (%)	11.0 ± 0.71	8.51 ± 0.69

## Data Availability

The data presented in this study are openly available in the Sydney eScholarship Repository at http://dx.doi.org/10.25910/2EVA-0569, accessed on 10 January 2022.
